# Conditional organs-of-interest segmentation with plausible inter-fraction variation simulation for pancreatic magnetic resonance-guided radiotherapy in limited-data settings

**DOI:** 10.1016/j.phro.2026.100973

**Published:** 2026-05-06

**Authors:** Mehdi Shojaei, Björn Eiben, Jamie R. McClelland, Simeon Nill, Alex Dunlop, Robert W. Chuter, Arabella Hunt, Brian Ng-Cheng-Hin, Uwe Oelfke

**Affiliations:** a Joint Department of Physics, Institute of Cancer Research and The Royal Marsden NHS Foundation Trust, London, UK; b The Royal Marsden NHS Foundation Trust, London, UK; c UCL Hawkes Institute and Department of Medical Physics and Biomedical Engineering, University College London, UK; d Christie Medical Physics and Engineering, The Christie NHS Foundation Trust, Manchester, UK; e Division of Cancer Sciences, School of Medical Sciences, Faculty of Biology, Medicine and Health, University of Manchester, Manchester, UK

**Keywords:** Magnetic resonance-guided radiotherapy, Pancreatic cancer, Deep-learning, Conditional segmentation, Data augmentation, Limited data

## Abstract

**Background and Purpose:**

Manual contouring of organs of interest (OoIs) is a major bottleneck in pancreatic magnetic resonance-guided online adaptive radiotherapy (oART). We developed C-SegDeform, a data-efficient conditional segmentation framework that used structure-guided deformation-based augmentations to simulate plausible inter-fraction anatomical variation and leveraged organ-specific conditioning as an alternative to registration-based contour propagation (Prop-ROIs) in limited-data settings.

**Materials and Methods:**

Forty balanced 3DVane images from 12 patients were manually contoured and pre-processed, including duodenum, both kidneys, liver, large and small bowel, spinal canal, spleen, and stomach. The training dataset (26 images) was augmented by simulating plausible session images via structure-guided deformations. Data was arranged for conditional segmentation and leave-one-out cross-validation using the nnU-Net framework. Analysis included geometric (DSC, average surface distance (ASD), and 95th percentile of Hausdorff distance (HD_95_); against Prop-ROIs and TotalSegmentator MRI), dose (via 8 treatment plans, measuring $D_{0.1\;cm^{3}}$ and $D_{50\text{\%}}$ discrepancies relative to prescribed dose), and clinical (Likert scale and post-auto-contour editing times by two oncology consultants) assessments.

**Results:**

C-SegDeform (DSC: 0.88±0.10, ASD: 2.9±2.4mm, HD_95_: 10.5±8.9mm) outperformed Prop-ROIs (0.70±0.17, 8.5±9.9mm, 16.7±9.8mm) and TotalSegmentator (0.75±0.16, 5.1±3.4mm, 14.3±10.6mm), and showed smaller relative dose deviations from ground-truth contours ($D_{0.1\;cm^{3}}$: 2.2%±2.5%, $D_{50\text{\%}}$: 0.5%±0.9%) than Prop-ROIs (6.8%±5.7%, 3.3%±4.0%). Clinicians ranked 98% of C-SegDeform contours as requiring no or minor edits (vs. 70% for Prop-ROIs), with editing times under 6 min and full OoI generation completed in under 40 s.

**Conclusions:**

C-SegDeform provided a robust alternative to contour propagation, generating accurate contours rapidly with minimal edits, thereby reducing on-couch time and streamlining the workflow.

## Highlights

• Conditional segmentation with guided deformations enhanced low-data generalizability.
• Mean Dice of 0.88 and surface distance of 2.9 mm for 9 organs across 14 test images.
• Median dose difference <0.5% for plans from C-SegDeform contours against ground truth.
• 98% of contours require no or minor edits, editing under 6 min and inference in 40 s.
• Significantly (*p* << 0.001) outperforming contour propagation and TotalSegmentator MR.

## Graphical abstract

**Figure:**
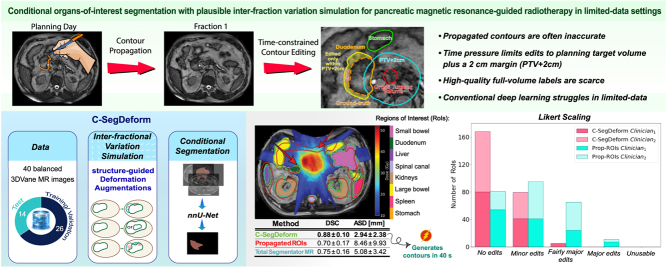

## 1 Introduction

Online adaptive radiotherapy (oART) for pancreatic cancer using magnetic resonance linear accelerators (MR-Linacs) enables high-precision treatment by accounting for daily anatomical variations. For effective adaptation, it is crucial to generate accurate contours on the session images quickly, as delays can lead to intra-fractional organ motion while the patient remains on the treatment couch. Registration-based contour propagation (Prop-ROIs) is currently used to transfer regions of interest from the offline image to the daily session images. Although useful as a starting point, these contours are often inaccurate and require substantial manual editing [1], [2]. To manage time constraints and reduce clinician workload, these edits are typically confined to the high-dose regions, defined as the planning target volume (PTV) plus a 2 cm margin (PTV+2 cm) [3], [4]. Nevertheless, the editing process usually exceeds the dedicated 15–20 min adaptation timeframe, supporting the need for more efficient and accurate solutions.

Artificial intelligence (AI)-based algorithms could significantly streamline this process, if they achieve sufficient accuracy to replace image registration within this workflow. In pancreatic MR-Linac radiotherapy, treatment session imaging often uses motion-robust 3D acquisitions such as 3DVane (3D stack-of-stars), which mitigate respiratory motion artefacts [5], [6] but typically sacrifice soft-tissue contrast. Here, 3DVane serves as a representative example of a common limitation across many clinical acquisitions: datasets are often small and annotations are inconsistently refined. Since available contours are typically modified only near the high-dose region under clinical time constraints, they are suboptimal for training robust AI models [7]. Moreover, auto-segmentation models might struggle with asymmetric anatomy (e.g., a single kidney). These cases increase anatomical variability and outliers, which is particularly challenging when training with limited data.

Patient-specific strategies have been proposed to mitigate these challenges. However, they typically require multiple training steps, substantially increasing workload and demanding expert oversight, and their performance/generalizability can still degrade across fractions when organ shape or intensity changes [8], [9], [10], [11]. Moreover, no commercially available auto-contouring solution is currently designed for MR-Linac 3DVane imaging, motivating the need for a dedicated AI-based framework tailored to magnetic resonance-guided adaptive workflows.

Across different radiotherapy fractions, the underlying anatomy of a patient typically remains consistent, with only localized changes occurring between sessions. This insight allows the use of initial offline images, together with their corresponding contour sets, to generate predictions for later fractions, without requiring patient-specific retraining. Leveraging this, we implemented C-SegDeform, a conditional segmentation approach [12] using the nnU-Net framework [7], [13], [14] and trained on data augmented using our previously developed structure-guided deformation-based augmentation (sgDefAug) method [7]. This approach simulated realistic anatomical variations across fractions, enabling the model to focus on spatially plausible changes specific to each patient. Our pipeline aimed to deliver fast and accurate OoI delineation suitable for integration into online adaptive workflows, with clinical relevance assessed via geometric performance and downstream clinical impact.

## 2 Materials and methods

### 2.1 Workflow

**Fig. 1:**
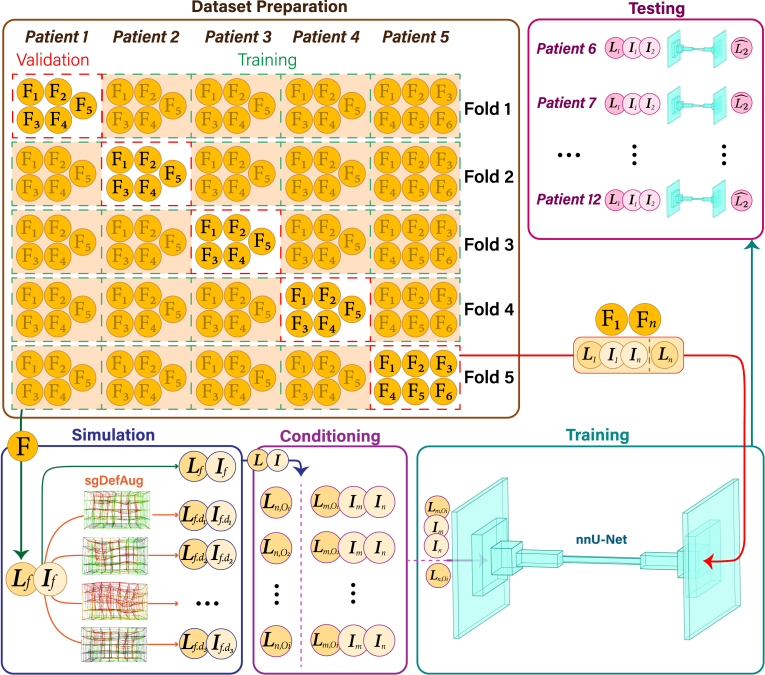
Flowchart of our workflow. After preparing and preprocessing the images and corresponding labels, these were input into the sgDefAug algorithm (simulation) to generate plausible session images. The simulated images were then conditioned and fed into the AI model for training. In dataset preparation, each row represents a separate fold, and each column corresponds to a patient, with one patient designated as the validation case in each fold. Patient 5 contributed six images, while the others contributed five. In addition to the cross-validation, an independent cohort of 7 test patients was used to evaluate the final model outputs. The symbols (*I*) and (*L*) denote the image and its corresponding labels, respectively. Additionally, (*f*), (*d*), and (*O*) represent each session (fraction) image, the simulated (deformed) image, and each OoI, respectively. The symbols (*m*) and (*n*) are used to represent two different images in the workflow.

The overall workflow is illustrated in Fig. 1. We began by preprocessing the dataset to ensure it is suitable for training. Next, we applied our previously developed sgDefAug algorithm to simulate plausible session images and corresponding contours, creating a diverse training set. We then incorporated a conditioning strategy within the nnU-Net framework to establish the segmentation model.

### 2.2 Data preparation

A total of 40 balanced 3DVane (b3DVane) images were acquired from 12 pancreatic cancer patients treated on a 1.5 T Unity MR-Linac. These anonymized images were obtained from the MOMENTUM study [15], approved under Research Ethics Committee reference 19/NW/0475 and Integrated Research Application System number 259162. Ethical approval was obtained at each participating center, and all patients provided written informed consent.

The dataset included nine OoIs essential for oART: duodenum, large and small bowels, liver, left and right kidneys, spleen, stomach, and spinal canal. Since the provided dataset contained edited Prop-ROIs only within the PTV+2 cm margin, manual contouring of all images was required from scratch. The manual contouring process was performed and independently validated by two experienced clinical oncology consultants. Each image required approximately 8 h to contour all OoIs. The preprocessing steps, including DICOM conversion, denoising, bias field correction, and normalization, were performed according to our previous work [7]. As offline planning images often differed in origin from session images, we reassigned the offline image/labels to the session geometry to ensure a common grid for network input.

A leave-one-out cross-validation strategy was implemented at the patient level to prevent data leakage, i.e, all session images from a given patient were confined to the same fold. Patients with 5 or 6 images (5 patients, 26 images) were used for training and validation, while patients with 2 images (7 patients, 14 ordered image pairs by evaluating both image directions) obtained prospectively were used for testing. The test cohort was intentionally selected to include challenging anatomical scenarios, such as patients with large cysts and asymmetric anatomy (e.g., a single kidney).

### 2.3 Simulation of inter-fraction anatomical variations

Plausible inter-fraction anatomical variability was simulated using our previously implemented algorithm, structure-guided deformation-based augmentations, applied to each session image with Platipy in Python [7], [16]. For each labeled OoI, random yet controlled displacement, scaling, and radial bending parameters were sampled from bounded uniform distributions to generate a plausible structure-specific deformation vector field (DVF).

In the notation of our method, let I(x) represent a session image and $L\left(x\right)=\left\{L_{1}\left(x\right),\ldots ,L_{N_{\text{OoI}}}\left(x\right)\right\}$ denote the corresponding set of labels, where each index corresponds to a distinct OoI. To simulate random deformations of a single label, a function ϕ is defined that takes a label and a fixed set of parameters that control the maximum amount of deformation applied to that particular label, comprising displacement, expansion, shrinkage, and rotation, i.e. $p=\left\{p_{\text{disp.}},p_{\text{exp.}},p_{\text{shrink.}},p_{\text{rot.}}\right\}$. Hence, the function ϕ creates a DVF u(x) as follows:

(1) $$u_{1}\left(x\right)=\phi \left(L_{1}\left(x\right),p\right)$$

To create a composed deformation that includes all labels, ϕ is applied iteratively to each label once:

(2) $$u_{N_{\text{OoI}}}\left(x\right)=\phi \left(L_{N_{\text{OoI}}}\left(u_{N_{\text{OoI}}-1}\circ \ldots \circ u_{1}\left(x\right)\right),p\right)$$

The final composed DVF $u_{\text{tot}}\left(x\right)=u_{N_{\text{OoI}}}\circ \ldots \circ u_{1}\left(x\right)$ can then be used to get a deformed instance of the session image and corresponding labels:

(3) $$I{\left(x\right)}^{\prime }=I\left(u_{\text{tot}}\left(x\right)\right)$$

(4) $$L{\left(x\right)}^{\prime }=\left\{L_{1}\left(u_{\text{tot}}\left(x\right)\right),\ldots ,L_{N_{\text{OoI}}}\left(u_{\text{tot}}\left(x\right)\right)\right\}$$

Note that each invocation of ϕ involves sampling a random variable from a uniform distribution, ensuring that the resulting deformation vector fields are unique even for identical input parameters.

In addition to sgDefAug, several MR-specific augmentations, including random histogram shifts, random intensity scaling, and random Gibbs noise were employed to better reflect realistic MRI acquisition variability between fractions.

### 2.4 Conditioning

To implement conditioning, images were concatenated to create three-channel input data ($D_{mn}$) from a total of N images (5). Here, the auto-segmentation model $S_{\theta }$, predicts the label L^n,o for the image $I_{n}$ using the image $I_{m}$, the corresponding label $L_{m,o}$ for the OoI o, and the image $I_{n}$ (6).

(5) $$D_{mn,o}=\left[I_{m},L_{m,o},I_{n}\right]\;\;\forall m,n\in \left\{1,2,\ldots ,N\right\}\;m\neq n\;\;\text{and}\;o\in \left\{1,2,\ldots ,N_{\text{OoIs}}\right\}$$

(6) L^n,o=Sθ(Dmn,o)

### 2.5 Training

Consistent with our prior results [7], [13], the nnU-Net framework was used. It auto-configures the architecture and key training settings from dataset ‘*fingerprints*’ (e.g., spacing, size, intensity, class balance), deriving patch/batch sizes and target spacing [17]. The loss function was nnU-Net’s default compound Dice similarity coefficient (DSC) and cross-entropy loss for multi-class segmentation. Further details on the model architecture are provided in Supplementary Section A.

By default, nnU-Net applies spatial (mirroring, elastic/affine transforms) and intensity augmentations (blurring, contrast/gamma changes, and low-resolution simulation). To avoid left–right kidney swaps, mirroring along the left–right axis was disabled.

The models were trained on a 24 GB NVIDIA RTX A5000 graphics processing unit (GPU) for 1500 epochs to reach convergence.

The code is not publicly available; it may be shared upon reasonable request, subject to institutional approvals.

### 2.6 Evaluation experiments

Geometric and dose-volume performance, statistical significance, and clinical impact were evaluated through clinician review, contour-quality scoring, and editing time to clinical acceptability.

To benchmark our approach, we compared C-SegDeform against the current clinical workflow (Prop-ROIs) and the open-source TotalSegmentator MRI [18]. Clinical validation was performed only against Prop-ROIs, as it represents the current workflow comparator, while TotalSegmentator was included as an open-source technical benchmark. For each method, outputs were generated using the first fraction’s image and labels ($I_{1},L_{1}$) to predict contours for subsequent fractions ($I_{2},\ldots ,I_{n}$).

Supplementary Section C further evaluates robustness by testing contour generation across arbitrary fraction pairs (C.1) and by exploring ensembling from multiple prior fractions (C.2).

### 2.7 Geometric analysis

DSC was used to quantify overlap, while boundary accuracy was assessed using the average surface distance (ASD) and the 95th percentile Hausdorff distance (HD_95_) [19], [20], [21].

### 2.8 Dose-volume analysis

For each patient, a session image was selected and two treatment plans were generated based on the unedited outputs of C-SegDeform and Prop-ROIs to evaluate the dosimetric consequences of the initial automated contour discrepancies. This analysis was not intended to represent the final clinical workflow, in which contours are reviewed and edited before use, but rather to determine whether differences in the automated starting contours could translate into clinically relevant dose deviations, particularly in high-dose-gradient regions. Treatment planning was performed using Elekta’s Research Monaco treatment planning system (version 6.09.01), with a prescription of 40 Gy to the PTV delivered in five fractions. The dose distributions derived from these plans were overlaid onto the ground-truth contours, and the dose differences for each structure were analyzed using dose-volume histograms (DVHs).

The DVH criteria for all nine OoIs were compared using D0.1cm3dPD and D50%dPD values. Here, d represents the difference between each method’s $D_{0.1\;{\mathrm{cm}}^{3}}$ and $D_{50\text{\%}}$ values and the corresponding ground-truth values, relative to the prescribed dose (PD). The $D_{0.1\;{\mathrm{cm}}^{3}}$ metric is sensitive to small hot spots and thus a good representation of the near-maximum dose received by an OoI. Furthermore, the modified Dice (mDice) metric [22], which incorporates the dose gradient as a weighting factor into the DSC, was also assessed. This metric represents the spatial relationship between an OoI and the target volume, giving a higher importance to those OoIs in closer proximity to the target.

### 2.9 Clinical validation: Likert scaling

Two experienced clinical oncology consultants independently evaluated clinical utility of the contours. Randomly selected contours were presented, with the clinicians blinded to their source. Each consultant ranked the contours using a customized Likert scale [23]; 1: no edits required, 2: minor edits required, 3: major edits required, but editing was preferable to starting from scratch, 4: major edits required and uncertainty whether to edit or start from scratch, and 5: unusable.

The clinicians also indicated their preference between C-SegDeform and Prop-ROIs for each OoI.

### 2.10 Clinical validation: Editing time

Automatic contours were edited to clinical acceptability. All edits were performed independently and in accordance with the clinical standards routinely applied in oART practice. Editing times were recorded to assess clinical applicability within these workflows, providing a direct estimate of potential time savings.

### 2.11 Statistical analysis

Paired comparisons were assessed using the Wilcoxon signed-rank test, with Holm’s sequential Bonferroni correction applied to control for multiple comparisons (α=0.05). Furthermore, a Spearman rank correlation test was applied to examine the relationship between the dose-volume criteria and the clinicians’ validation scores.

## 3 Results

Training took approximately 5 days per fold and the inference was around 5 s per OoI (<40 s total). Given the limited dataset size, validation-set results are reported separately in Supplementary Section B

C-SegDeform achieved higher DSC across OoIs (0.88 ± 0.10) than Prop-ROIs (0.70 ± 0.17) and TotalSegmentator MRI (0.75 ± 0.16) (Table 1); TotalSegmentator was comparable for stomach DSC and had lower distance errors. Mean ASD was lower with C-SegDeform (2.9 ± 2.4 mm) than Prop-ROIs (8.5 ± 9.9 mm) and TotalSegmentator (5.1 ± 3.4 mm). HD_95_ was also lower for C-SegDeform (10.7 ± 8.9 mm) than for Prop-ROIs (16.7 ± 9.8 mm) and TotalSegmentator (14.3 ± 10.6 mm).

Dose analysis (Table 2) showed that C-SegDeform yielded lower dose discrepancies than Prop-ROIs and aligned more closely with the ground truth (p=0.0003 for D0.1cm3dPD and p=0.0004 for D50%dPD). For C-SegDeform, 44% and 56% of OoIs had deviations below 1% for $D_{0.1\;{\mathrm{cm}}^{3}}$ and $D_{50\text{\%}}$, compared with 22% and 33% for Prop-ROIs. Deviations above 5% occurred in 33% and 11% of cases for C-SegDeform, versus 56% and 33% for Prop-ROIs. Discrepancies were greatest for OoIs close to the PTV, such as the small bowel, duodenum, and stomach. mDice also favored C-SegDeform (0.91 vs 0.75), supporting its value as a target-focused complement to DSC.

**Table 1:** A comparison of the performance between C–SegDeform, Prop–ROIs, and TotalSeg(mentator MRI) across 14 test images. Higher accuracy (higher DSC, lower ASD and HD_95_) is highlighted in bold. Statistical significance is reported to the right of each method comparison for each metric; p-values are Holm-adjusted for multiple comparisons, with a bracket and asterisks denoting * p<0.05, ** p<0.01, *** p<0.001.

**Fig. 2:**
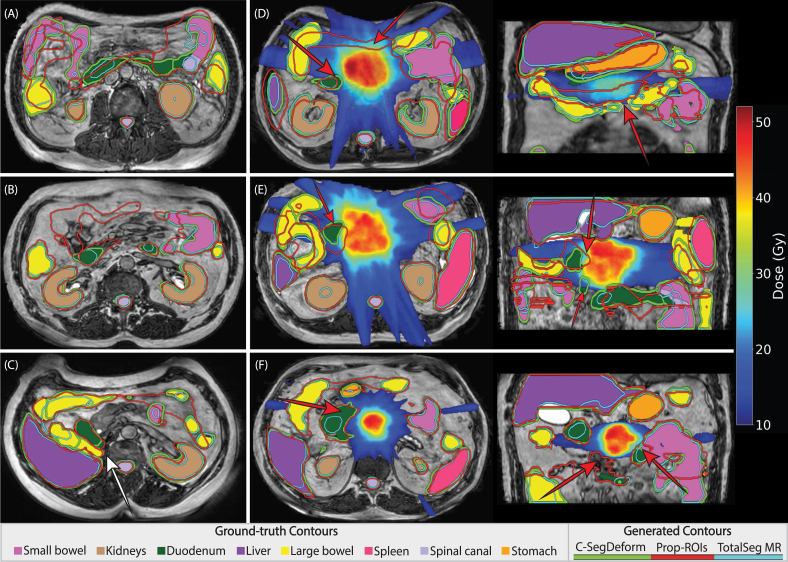
Comparison between the contours from C-SegDeform, Prop-ROIs and TotalSegmentator MRI. Panels (A), (B), and (C) demonstrate the contour outputs superimposed on the ground-truth contours (filled) for three patients. Panel (C) illustrates a case with only one kidney; the white arrow indicates the location where the right kidney would normally appear. Substantial discrepancies are observed between the Prop-ROIs/TotalSegmentator MRI and the ground-truth contours, specifically in the bowels. Panels (D), (E), and (F) show the contours with dose distribution overlay acquired from Prop-ROIs for different patients along with C-SegDeform outputs, revealing that the Prop-ROIs have substantial inaccuracies in high-dose gradient regions, leading to erroneous dose assessment and failure to identify OoI overdosage leading to longer editing times in an oART workflow. Red arrows indicate the large errors.

Fig. 2 supports these findings; near the target (D–F), Prop-ROIs misalignment translates into larger dose discrepancies (up to 40 Gy), whereas C-SegDeform remains aligned in high-dose regions.

**Fig. 3:**
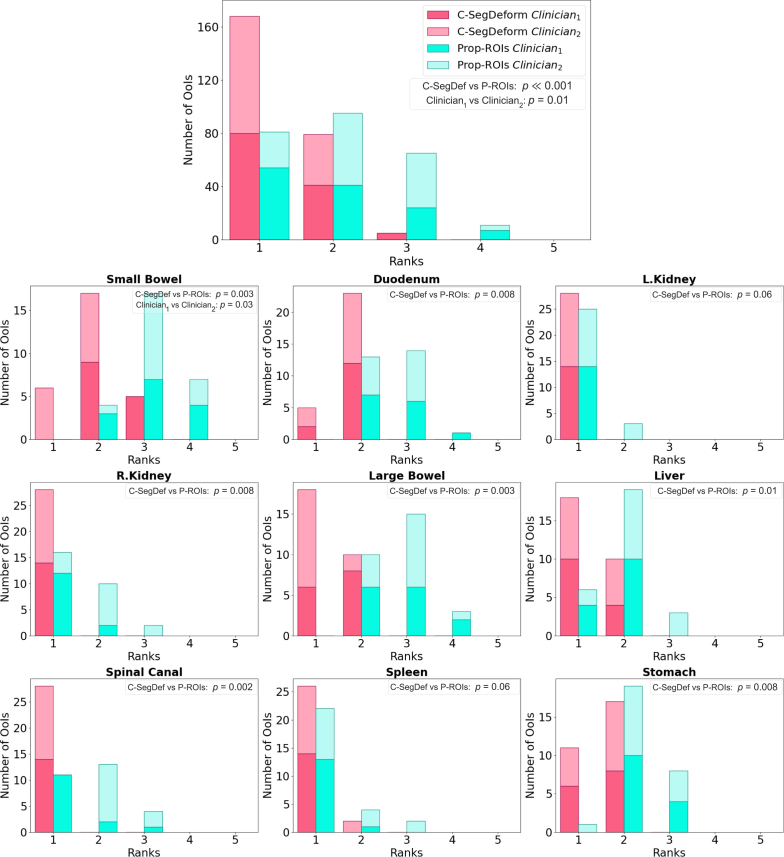
Likert ranks comparing contour quality across OoIs. The first row shows the scores across all the OoIs. Two experienced clinical oncology consultants independently evaluated the generated contours. Statistical significance is indicated in the figure legend for comparisons between methods and between clinicians.

Likert ranks (Fig. 3) clustered at lower scores for C-SegDeform (fewer edits) and higher scores for Prop-ROIs, with a median paired difference of −1.0 [−1.5 to −1.0]. Clinician 1 scored approximately 96% of C-SegDeform contours as 1 (no edits required) or 2 (minor edits required), with no ranks of 4 or 5, while clinician 2 assigned ranks of 1 or 2 to all C-SegDeform contours. For Prop-ROIs, clinician 1 ranked 25% and clinician 2 ranked 36% of contours as 3 or 4.

**Table 2:** A comparison of the performance between C-SegDeform and Prop-ROIs across 4 treatment plan pairs from 4 patients. In D0.1cm3dPD and D50%dPD, d denotes the difference between each method’s $D_{0.1\;cm^{3}}$ and $D_{50\text{\%}}$ values and the corresponding ground-truth values, relative to the prescribed dose (PD). Higher accuracy (higher DSC/mDice and lower dose discrepancy) is highlighted in bold. The additional DSC column reports DSC for the subset of cases used to compute mDice.

Organs	D0.1cm3dPD [%]		D50%dPD [%]		mDice		DSC (mDice Subset)	
	C-SegDef	Prop-ROIs	C-SegDef	Prop-ROIs	C-SegDef	Prop-ROIs	C-SegDef	Prop-ROIs
Liver	7.8±12.7	7.9±5.2	1.4±1.9	1.7±0.8	0.94±0.04	0.87±0.04	0.97±0.03	0.90±0.04
Duodenum	5.6±4.9	10.0±8.0	4.8±4.9	9.1±9.2	0.80±0.08	0.72±0.10	0.70±0.18	0.61±0.09
L. Kidney	0.2±0.2	0.8±0.5	1.2±2.0	1.9±1.1	0.94±0.05	0.89±0.05	0.97±0.02	0.88±0.04
R. Kidney	0.6±0.4	1.5±1.7	0.2±0.2	0.8±0.9	0.97±0.00	0.90±0.04	0.98±0.00	0.84±0.03
Spinal Canal	0.2±0.2	0.9±0.8	0.0±0.0	2.4±1.1	0.98±0.01	0.85±0.05	0.98±0.02	0.69±0.12
Spleen	0.5±0.6	1.8±2.0	0.4±0.6	0.5±0.3	0.96±0.03	0.87±0.08	0.88±0.13	0.85±0.07
Small Bowel	1.6±1.2	13.1±11.1	0.3±0.4	8.9±6.5	0.86±0.09	0.50±0.05	0.72±0.13	0.46±0.12
Large Bowel	4.0±4.9	19.8±8.9	7.6±12.5	12.8±10.2	0.90±0.04	0.55±0.18	0.75±0.23	0.53±0.15
Stomach	15.1±8.9	24.9±15.0	0.3±0.4	0.5±0.2	0.83±0.04	0.60±0.13	0.76±0.17	0.60±0.25

Mean	4.0±4.3	9.0±4.9	1.8±3.8	4.3±3.9	0.91±0.08	0.75±0.05	0.86±0.18	0.71±0.20

Across OoIs, C-SegDeform was typically ranked 1–2; small bowel was the only exception (occasional 3), while Prop-ROIs was generally ranked 2–4, especially for bowel structures. Both clinicians preferred C-SegDeform over Prop-ROIs for every OoI.

Likert scores correlated most strongly with mDice (Spearman r=−0.70), and less strongly with dose-discrepancy metrics (D50%dPD: r=0.66; D0.1,cm3dPD: r=0.46).

Table 3 summarizes the time required to edit C-SegDeform outputs for online adaptive pancreatic cancer SBRT. The mean total editing time per patient was under 6 min. Both kidneys required no modifications, while the duodenum required the longest editing time. Other abdominal OoIs generally required only minor refinements, which were typically completed in under 1 min.

**Table 3:** Time required to edit C-SegDeform outputs for online adaptive pancreatic cancer SBRT, performed by two clinical oncology consultants across all test cases.

Organs	Editing time [s]
Liver	7±16
Duodenum	198±176
L. Kidney	20±45
R. Kidney	0
Spinal Canal	0
Spleen	14±31
Small Bowel	47±59
Large Bowel	40±89
Stomach	28±45

Total	354±58

## 4 Discussion

This study showed that C-SegDeform outperformed contour propagation for pancreatic oART in geometric accuracy, dose similarity, and clinical validation, despite the limited dataset. This performance likely arose from combining localized spatial guidance through conditional segmentation and simulated anatomical variations. By focusing on smaller predefined regions for each OoI, the model achieved greater precision.

Prop-ROIs inaccuracies (Fig. 2 (D–F)) occasionally extended into high-dose gradient regions. This was further reflected in our clinical validation using customized Likert scaling (Fig. 3), where Prop-ROIs required substantial edits, while C-SegDeform often required no edits for rigid OoIs (kidneys, spinal canal) and only slight modifications for more challenging structures such as the small bowel. Editing-time analysis further supported these findings (<6 min for editing; <40 s for inference) suggesting that the proposed workflow may streamline oART and reduce on-couch time. Shorter sessions may also reduce intra-fraction anatomical changes, improving agreement between delivered and planned dose. Nevertheless, Prop-ROI editing times were not recorded, so direct time savings cannot be quantified. Another limitation was that TotalSegmentator was not clinically evaluated; therefore, we cannot comment on its editing burden or clinical acceptability in this workflow.

The duodenum required longer editing times, likely because it is commonly located within the PTV+2 cm margin and therefore requires greater care during review and editing. Finally, while C-SegDeform outperformed Prop-ROIs and TotalSegmentator for most OoIs, its performance on the stomach was lower or comparable. This likely relates to the single-label strategy under partial field-of-view, and indistinct duodenum–stomach boundary, which can increase misclassification. In cases where the stomach was fully captured, these issues were mitigated.

Although auto-contouring for some anatomical sites on the MR-Linac has been widely studied [24], [25], pancreatic cancer remains underexplored. To the best of our knowledge, this is the first study to investigate auto-segmentation of all clinically relevant OoIs for pancreatic cancer treatment planning using MR-Linac images, with results validated from a clinical perspective. In our previous work [7], we achieved a mean DSC of 0.84 ± 0.09, and ASD of 3.1 ± 1.8 mm for the best-performing fold, across all OoIs, excluding the large bowel. The current study demonstrated improvements, with a mean DSC of 0.88 ± 0.10 and an ASD of 2.9 ± 2.3 mm, while including the large bowel. Unlike our previous work and most other methods, which often failed with asymmetrical anatomies or missing organs, C-SegDeform achieved high accuracy (as shown in Fig. 2.C) with limited datasets while accommodating anatomical variations, as it was conditioned on each OoI individually, rather than processing all structures simultaneously. Specifically, challenging anatomical cases were included, such as patients with a missing kidney and polycystic organs with high-intensity cystic regions, to stress-test the pipeline. Nonetheless, future evaluation on a larger cohort, including more asymmetric cases, will be valuable to further validate and generalize these findings.

Alternative strategies have explored patient-specific models trained on an initial image to generate contours for the subsequent fractions [8], [11]. De Benetti et al. incorporated planning contours into dual-channel/encoder nnU-Net models without requiring retraining, achieving a mean DSC of 0.86 across eight abdominal OoIs [26]. However, their approach excluded several clinically important OoIs for pancreatic oART, such as the large bowel and spleen. Kawula et al. employed a per-patient fine-tuning strategy that required approximately one hour of training per case [10], substantially increasing workload and demanding expert oversight, while limiting generalizability. A key strength of our approach is achieving higher geometric accuracy despite being trained on a substantially smaller cohort. Our single-label conditional formulation with a three-channel input provides spatial guidance that is particularly beneficial for asymmetric and highly deformable OoIs relevant to pancreatic SBRT. Our results indicate that simulating plausible inter-fraction anatomical variation through augmentation can deliver robust performance without the additional complexity of patient-specific retraining.

Although our framework required prior contours as input, these are readily available from the reference planning scan. Notably, both contour propagation and patient-specific approaches also depend on prior manual contours. In practice, C-SegDeform can be integrated as a drop-in OoI auto-contouring step by using the available reference contours as conditioning input and generating session OoI contours for clinician review.

While our study demonstrated the feasibility of C-SegDeform using a limited dataset, a key limitation was the dataset size. This was unavoidable due to the time-intensive process of fully contouring all OoIs and the lack of high-quality annotations in existing datasets, which often contain only partial edits near the target volume. Therefore, a more comprehensive evaluation on a larger, independent cohort is essential to assess the method’s generalizability and robustness. C-SegDeform may also extend to other sites and image contrasts where only small, high-quality annotated datasets are available (e.g., liver), particularly when OoIs overlap with pancreatic anatomy. However, this requires further investigation, and future work should assess its generalizability beyond the abdominal region. Additionally, this study only focused on OoIs and did not address target delineation. Targets are typically propagated from the reference plan and edited, often using additional imaging; extending the framework to targets is left for future work.

C-SegDeform showed strong potential to reduce clinician workload and treatment time, enabling higher throughput in busy clinical settings. Clinical validation further underscored its readiness for adoption, with more than 98% of contours ranked as requiring minor or no edits. By generating accurate contours rapidly, C-SegDeform mitigates risks associated with intra-fraction anatomical changes and ensures consistent, precise dose delivery.

## CRediT authorship contribution statement

**Mehdi Shojaei:** Writing – review & editing, Writing – original draft, Visualization, Validation, Software, Resources, Project administration, Methodology, Investigation, Formal analysis, Data curation, Conceptualization. **Björn Eiben:** Writing – review & editing, Writing – original draft, Supervision, Project administration, Methodology. **Jamie R. McClelland:** Writing – review & editing, Supervision, Project administration, Methodology. **Simeon Nill:** Writing – review & editing, Supervision, Project administration, Methodology, Funding acquisition, Conceptualization. **Alex Dunlop:** Writing – review & editing, Validation, Supervision, Methodology. **Robert W. Chuter:** Writing – review & editing, Validation, Data curation. **Arabella Hunt:** Writing – review & editing, Validation, Methodology, Data curation. **Brian Ng-Cheng-Hin:** Writing – review & editing, Validation, Methodology, Data curation. **Uwe Oelfke:** Writing – review & editing, Supervision, Resources, Project administration, Methodology, Funding acquisition, Conceptualization.

## Declaration of competing interest

The authors declare that they have no known competing financial interests or personal relationships that could have appeared to influence the work reported in this paper.

## Appendix A Supplementary data

MMC S1 Supplementary model design and validation analyses for C-SegDeform, including cross-validation, random-fraction and ensemble tests. MMC S1
