# Supplementary material for: Conditional organs-of-interest segmentation with plausible inter-fraction variation simulation for pancreatic magnetic resonance-guided radiotherapy in limited-data settings
Source: Phys Imaging Radiat Oncol. 2026 May 6;39:100973. doi: 10.1016/j.phro.2026.100973 (PMC13196127; doi:10.1016/j.phro.2026.100973)
Supplement: MMC S1 — Supplementary model design and validation analyses for C-SegDeform, including cross-validation, random-fraction and ensemble tests. [file mmc1.pdf]

Supporting Material

In this Supplementary Material, we have provided detailed information on the model architecture and training configuration, reported the cross-validation results (given the limited dataset size), and outlined additional validation analyses that was performed on the cross-validation cohort.

A Model Design

The network used in our study was the standard 3-D full-resolution nnU-Net. It follows a U-shaped encoder-decoder design with five resolution levels. At the highest resolution, the network processes patches of $64 \times 192 \times 192$ voxels (depth x height x width) and produces 32 feature channels after the first convolution. Each encoder level contains two $3 \times 3 \times 3$ convolutions followed by instance normalization and a leaky-ReLU activation. Downsampling is achieved via strided $3 \times 3 \times 3$ convolutions (stride 2) that halve the spatial resolution and double the number of channels ($32 \rightarrow 64 \rightarrow 128 \rightarrow 256 \rightarrow 320$). The decoder mirrors the encoder. Feature maps from the encoder are concatenated via skip connections, spatial resolution is restored using transposed convolutions, and the number of channels is reduced back to 32 before the final $1 \times 1 \times 1$ convolution produces the segmentation logits.
Stochastic gradient descent (SGD) was used as the optimiser, with an initial learning rate of 1×10^{-2}, momentum of 0.99, and weight decay of 3×10^{-5}. The loss function combined DSC-based and cross-entropy loss terms. The patch size and mini-batch size were automatically determined by nnU-Net's fingerprinting process based on the median resampled image shape. These details are consistent with the framework's standard automated parameter selection procedure, as described in [1].

Using sgDefAug, four images were simulated from each session image. For each patient in the training set (folds 1 to 4), which included five session images (except for patient 5, which had six), a total of 20 images were simulated. When combined with the original session images, this resulted in 25 images per patient, except for patient 5, which had 30 images. Under the conditional training strategy, as outlined in (2.5) and (2.6), the total input for training can be calculated. For example, with 9 OoIs and 4 patients (each contributing 25 images), the total training dataset size for each fold was $P(25, 2) \times 9_{\text{OoIs}} \times 4_{patients} = 21600$, where $P(n, k)$ is the number of k permutations of n, which calculates the number of ways to arrange 2 objects out of 25 in a specific order.

B Cross-validation Results

Figure S1 shows the scatter plots comparing the geometric accuracy of C-SegDeform and Prop-ROIs. The contours generated by C-SegDeform were predominantly clustered closer to the bottom-right corner of the plot, indicating higher DSC values and lower ASD. In contrast, Prop-ROIs were distributed in regions associated with worse alignment. C-SegDeform demonstrated superior performance across all OoIs, as further evidenced by the quantitative results presented in Table S1, averaged over all folds of the validation set. The mean ASD for C-SegDeform outputs was approximately four times smaller than that of Prop-ROIs and TotalSegmentator MRI (1.5±1.4 mm vs. 6.1±6.3 mm and 5.4±5.4 mm, respectively). Similarly, the mean DSC improved from 0.75±0.16 for Prop-ROIs and 0.76±0.15 for TotalSegmentator MRI to 0.94±0.05 for C-SegDeform. A comparable trend was observed for HD_{95}, where C-SegDeform achieved values approximately one-third those of Prop-ROIs and TotalSegmentator MRI (6.0±5.9 mm vs. 16.0±14.8 mm and 16.0±16.1 mm, respectively).

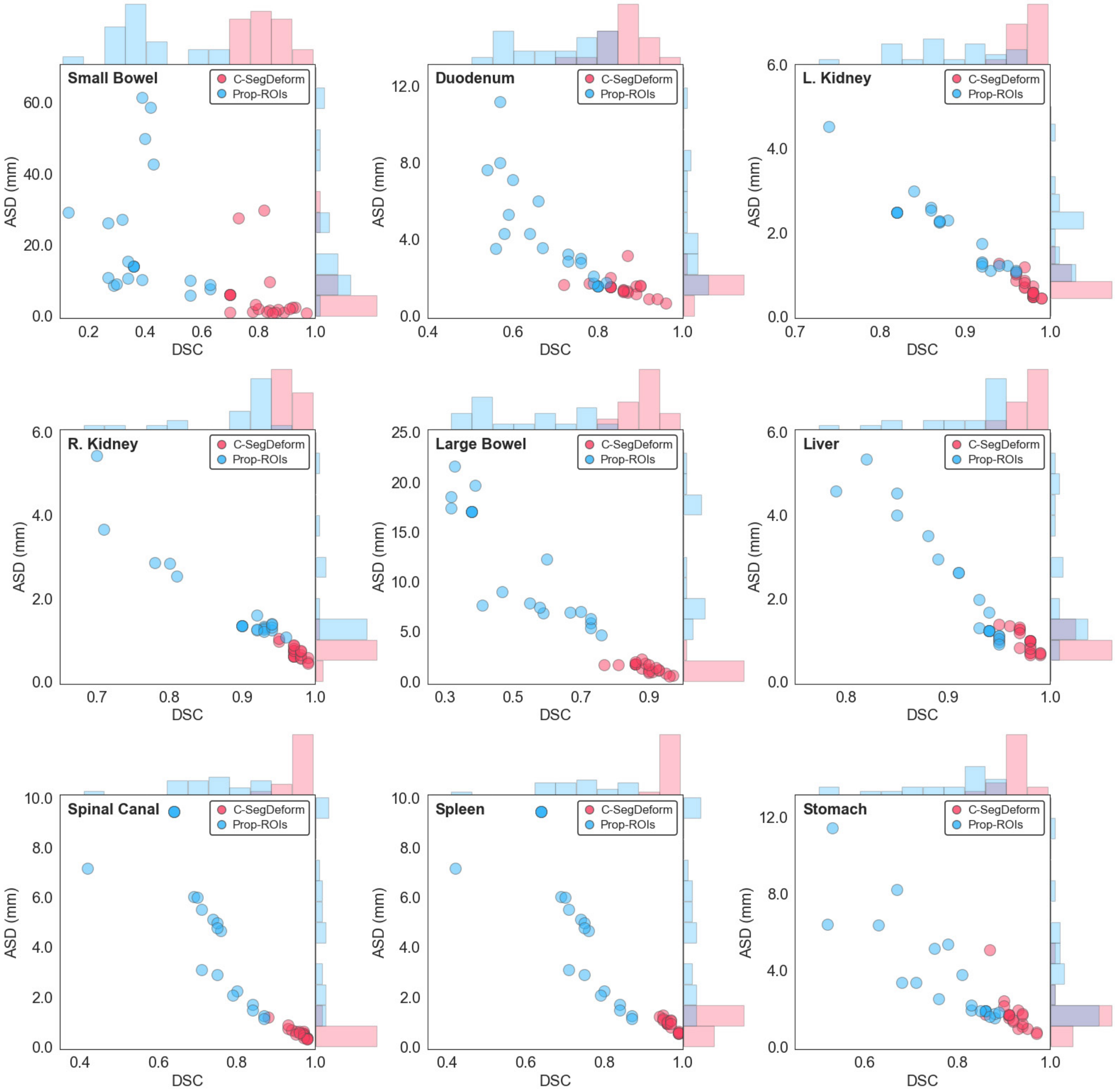

Figure S1: *Comparison between the contours from C-SegDeform and Prop-ROIs with DSC on the x-axis and ASD on the y-axis for each OoI. C-SegDeform's outputs are tightly clustered in the optimal performance region (high DSC values and low ASD). This concentrated distribution indicates that the contours generated by C-SegDeform closely match the ground-truth.*

Table S2 presents the $D^{d/PD}_{0.1\,cm^3}$, $D^{d/PD}_{50\,\%}$, and mDice metrics, where d represents the dose difference between each metric when overlaid onto the ground-truth contours, and PD denotes the prescribed dose (40 Gy). The results indicated that C-SegDeform had lower discrepancies in dose criteria compared to Prop-ROIs, demonstrating closer alignment to the ground-truth. Specifically, nearly 56 % of OoIs showed deviations of less than 1 % in near-maximum dose ($D_{0.1\,cm^3}$), and 89 % of OoIs exhibited deviations of less than 1 % in median dose ($D_{50\,\%}$) when using C-SegDeform contours relative to the prescribed dose. In contrast, for Prop-ROIs, only 22 % of OoIs had relative deviations of less than 1 % for $D_{0.1\,cm^3}$, and 56 % showed relative deviations of less than 1 % for $D_{50\,\%}$. Moreover, Prop-ROIs presented relative deviations exceeding 5 % in 56 % and 33 % of cases for $D_{0.1\,cm^3}$ and $D_{50\,\%}$, respectively, whereas C-SegDeform showed such relative deviations in only 22 % and 0 % of cases. On average, the mean $D^{d/PD}_{0.1\,cm^3}$ for Prop-ROIs was 6.8 %, meaning that, for a prescribed dose of 40 Gy, the discrepancy across all OoIs was approximately 2.7 Gy. In contrast, the discrepancy for C-SegDeform was about one-third of

Table S1: *A comparison of the performance between C–SegDeform, Prop–ROIs, and TotalSeg(mentator MRI) across 26 images from 5 patients (mean fold from cross-validation). Higher accuracy (higher DSC, lower ASD and HD_{95}) is highlighted in bold.*

Organs	DSC			ASD [mm]			HD_{95} [mm]		
	C–SegDef	Prop–ROIs	TotalSeg	C–SegDef	Prop–ROIs	TotalSeg	C–SegDef	Prop–ROIs	TotalSeg
Liver	$\mathbf{0.98 \pm 0.02}$	0.92 ± 0.05	0.91 ± 0.02	$\mathbf{1.0 \pm 0.3}$	2.1 ± 1.4	3.0 ± 0.6	$\mathbf{3.0 \pm 1.5}$	7.1 ± 5.4	10.2 ± 2.7
Duodenum	$\mathbf{0.87 \pm 0.06}$	0.70 ± 0.10	0.43 ± 0.12	$\mathbf{1.5 \pm 0.5}$	3.9 ± 2.6	19.6 ± 12.8	$\mathbf{6.4 \pm 2.9}$	13.9 ± 9.3	56.8 ± 38.6
L. Kidney	$\mathbf{0.98 \pm 0.02}$	0.88 ± 0.06	0.86 ± 0.02	$\mathbf{0.7 \pm 0.3}$	2.1 ± 0.8	1.9 ± 0.3	$\mathbf{1.9 \pm 0.6}$	6.8 ± 3.8	6.0 ± 0.9
R. Kidney	$\mathbf{0.98 \pm 0.02}$	0.89 ± 0.08	0.88 ± 0.02	$\mathbf{0.7 \pm 0.2}$	1.8 ± 1.1	1.7 ± 0.3	$\mathbf{1.7 \pm 0.4}$	4.6 ± 2.9	5.0 ± 0.9
S. Canal	$\mathbf{0.96 \pm 0.03}$	0.80 ± 0.11	0.89 ± 0.03	$\mathbf{0.5 \pm 0.2}$	4.9 ± 2.9	0.9 ± 0.3	$\mathbf{1.0 \pm 0.2}$	8.0 ± 5.9	2.0 ± 0.4
Spleen	$\mathbf{0.97 \pm 0.02}$	0.86 ± 0.08	0.87 ± 0.04	$\mathbf{0.9 \pm 0.3}$	2.8 ± 1.2	2.7 ± 0.6	$\mathbf{2.6 \pm 1.0}$	7.8 ± 3.5	5.9 ± 1.8
S. Bowel	$\mathbf{0.82 \pm 0.09}$	0.40 ± 0.13	0.57 ± 0.18	$\mathbf{5.4 \pm 8.8}$	21.8 ± 17.1	7.0 ± 3.8	$\mathbf{20.8 \pm 14.8}$	53.0 ± 19.8	22.4 ± 11.5
L. Bowel	$\mathbf{0.90 \pm 0.05}$	0.52 ± 0.16	0.68 ± 0.14	$\mathbf{1.4 \pm 0.5}$	11.6 ± 5.6	7.3 ± 5.2	$\mathbf{6.3 \pm 4.0}$	30.9 ± 11.1	23.7 ± 21.1
Stomach	$\mathbf{0.93 \pm 0.03}$	0.78 ± 0.12	0.79 ± 0.08	$\mathbf{1.6 \pm 1.0}$	3.5 ± 2.6	4.3 ± 1.9	$\mathbf{9.9 \pm 10.0}$	12.1 ± 6.1	11.9 ± 5.1
Mean	$\mathbf{0.94 \pm 0.05}$	0.75 ± 0.16	0.76 ± 0.15	$\mathbf{1.5 \pm 1.4}$	6.1 ± 6.3	5.4 ± 5.4	$\mathbf{6.0 \pm 5.9}$	16.0 ± 14.8	16.0 ± 16.1

Table S2: *A comparison of the performance between C-SegDeform and Prop-ROIs across 5 treatment plan pairs from 5 patients. In $D^{d/PD}_{0.1\,cm^3}$ and $D^{d/PD}_{50\,\%}$, d denotes the difference between each method's $D_{0.1\,cm^3}$ and $D_{50\,\%}$ values and the corresponding ground-truth values, relative to the prescribed dose (PD). Higher accuracy (higher mDice and lower dose discrepancy) is highlighted in bold.*

Organs	$D^{d/PD}_{0.1\,cm^3}$ [%]		$D^{d/PD}_{50\,\%}$ [%]		mDice	
	C-SegDef	Prop-ROIs	C-SegDef	Prop-ROIs	C-SegDef	Prop-ROIs
Liver	$\mathbf{3.8 \pm 3.9}$	5.8 ± 6.3	$\mathbf{0.0 \pm 0.0}$	0.1 ± 0.1	$\mathbf{0.95 \pm 0.02}$	0.89 ± 0.05
Duodenum	$\mathbf{6.8 \pm 8.7}$	10.0 ± 7.7	$\mathbf{2.9 \pm 2.0}$	6.7 ± 9.7	$\mathbf{0.90 \pm 0.03}$	0.77 ± 0.13
L. Kidney	$\mathbf{0.4 \pm 0.2}$	0.9 ± 0.5	$\mathbf{0.1 \pm 0.0}$	0.8 ± 1.0	$\mathbf{0.98 \pm 0.01}$	0.91 ± 0.06
R. Kidney	$\mathbf{0.4 \pm 0.4}$	1.5 ± 1.2	$\mathbf{0.1 \pm 0.1}$	0.5 ± 0.7	$\mathbf{0.98 \pm 0.01}$	0.91 ± 0.05
Spinal Canal	$\mathbf{0.0 \pm 0.0}$	0.9 ± 1.1	$\mathbf{0.0 \pm 0.0}$	2.5 ± 2.6	$\mathbf{0.98 \pm 0.01}$	0.87 ± 0.07
Spleen	$\mathbf{0.1 \pm 0.1}$	1.4 ± 2.1	$\mathbf{0.0 \pm 0.0}$	0.2 ± 0.2	$\mathbf{0.98 \pm 0.01}$	0.89 ± 0.08
Small Bowel	$\mathbf{2.5 \pm 0.7}$	12.7 ± 10.8	$\mathbf{0.5 \pm 0.4}$	12.3 ± 10.2	$\mathbf{0.82 \pm 0.10}$	0.59 ± 0.18
Large Bowel	$\mathbf{0.3 \pm 0.3}$	10.5 ± 9.7	$\mathbf{1.0 \pm 1.4}$	6.2 ± 5.0	$\mathbf{0.91 \pm 0.06}$	0.63 ± 0.23
Stomach	$\mathbf{5.7 \pm 6.0}$	17.2 ± 15.3	$\mathbf{0.1 \pm 0.0}$	0.2 ± 0.2	$\mathbf{0.86 \pm 0.07}$	0.67 ± 0.19
Mean	$\mathbf{2.2 \pm 2.5}$	6.8 ± 5.7	$\mathbf{0.5 \pm 0.9}$	3.3 ± 4.0	$\mathbf{0.93 \pm 0.06}$	0.79 ± 0.12

that value. Organs in close proximity to the PTV, such as the small bowel, duodenum, and stomach, had higher discrepancies. While mDice also favoured C-SegDeform, the slightly smaller difference compared to standard DSC indicated that incorporating dose distribution reduced the big performance gap between C-SegDeform and Prop-ROIs.

As shown in Figure S2 the contours generated by C-SegDeform are predominantly distributed toward the left side of the chart, indicating fewer required edits, while Prop-ROIs are more centrally distributed, suggesting a greater need for modifications. Clinician 1 scored approximately 95 % of the C-SegDeform contours as either 1 (no edits required) or 2 (minor edits required), with no ranks of 4 or 5. Similarly, Clinician 2 assigned ranks of 1 or 2 to all C-SegDeform contours, except for only one case. On the other hand, clinician 1 scored 27 % and clinician 2 scored 34 % of Prop-ROIs as 3 or 4.

Across OoIs, both clinicians predominantly ranked C-SegDeform contours as 1 (no edits required) or 2 (minor edits), indicating minimal need for adjustments across most OoIs. The small bowel was the only exception, with some cases receiving a score of 3 (major edits required, but preferable to starting from scratch). In contrast, Prop-ROI rankings skewed higher (2 to 4), particularly for deformable structures such as the small and large bowels, reflecting a greater need for manual corrections. The best performance was observed for OoIs located farther from the target, where the dose gradient is lower.

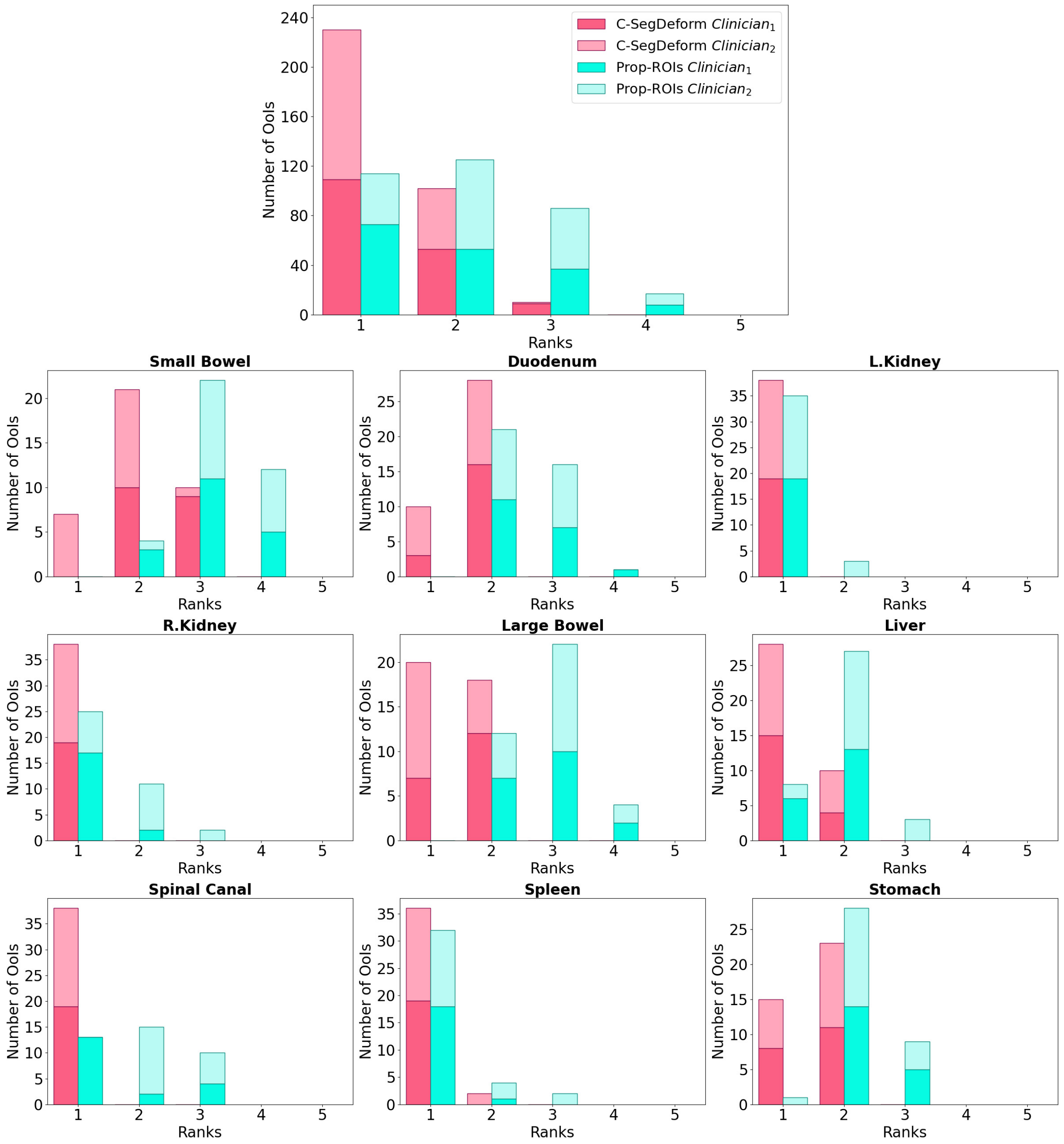

Figure S2: *Comparison between the ranks from the Likert scaling system to assess the generated contours across each OoI. The first row shows the scores across all the OoIs. Two experienced clinical oncology consultants independently evaluated the contours.*

Both clinicians were also asked to indicate their preference between C-SegDeform and Prop-ROIs for the same anatomical structures. In all cases, they selected C-SegDeform over Prop-ROIs.

C Further Validation

To rigorously evaluate the generalizability and robustness of our proposed framework, we conducted additional testing beyond the conventional first-fraction-to-subsequent-fractions workflow. This analysis serves two key purposes:

1. **Comprehensive Input Validation:** To validate the model's ability to generate contours from any prior fraction (not limited to the first fraction) for subsequent sessions, simulating

real-world scenarios where anatomical changes may make earlier fractions less optimal as references as well as introducing more test inputs.

2. **Ensemble Learning Potential:** To explore whether aggregating predictions from multiple prior fractions improves contour accuracy, leveraging inter-fraction anatomical correlations.

C.1 Comprehensive Random Input Validation

While our standard workflow for validation uses an initial image (I_1,L_1) to predict contours for subsequent fractions (I_2,...,I_n), we extended this to allow any fraction as input (e.g., generating L_5 from I_3 or I_4 or vice versa). This flexibility serves two purposes: it expands the variety of test inputs for the AI model and accounts for the fact that later fractions may better reflect anatomical states due to shorter intervals and improved positional reproducibility. For example, generating L_5 from I_4 rather than I_1 may yield better results if anatomical differences between fractions 4 and 5 are smaller than those between 1 and 5.

Table S3 summarizes the outputs of this evaluation. Specifically, we evaluated whether the time interval between fractions influenced contour accuracy by generating contours for I_5 and I_4 using each of the preceding fractions as prior inputs. The resulting accuracies were comparable across different fraction intervals, suggesting that using a temporally closer prior fraction did not consistently yield improved performance.

Furthermore, a systematic evaluation of any random fraction pairs (I_m and I_n) was performed to quantify performance variations on more test data. As illustrated in Figure S3, the outputs conditioned on random fractions confirmed that C-SegDeform consistently outperformed Prop-ROIs, with its contours clustering toward regions of higher DSC values (closer to 1) and lower ASD (closer to 0). It is worth mentioning that the Prop-ROI results were not permuted and were identical to those shown in the main body.

Table S3: *Comparison between the geometric accuracy results from the impact of using different fraction intervals and random fraction pairs as inputs for contour generation. The results indicate no significant correlation between the fraction interval (i.e., the temporal distance between the input and target fractions) and contour accuracy. This suggests that using closer fractions does not necessarily improve performance. The results from random fraction pairs indicate that the model is robust to variations in input fraction selection and can reliably generate accurate contours regardless of the specific fraction used as input.*

Organs	DSC					ASD [mm]				
	Random Fractions	Fraction Intervals				Random Fractions	Fraction Intervals			
		1	2	3	4		1	2	3	4
Small Bowel	0.84	0.82	0.85	0.82	0.86	3.5	4.3	3.7	3.6	6.2
Duodenum	0.87	0.87	0.89	0.88	0.88	1.8	1.6	1.6	1.8	1.8
L. Kidney	0.98	0.98	0.98	0.98	0.97	0.7	0.7	0.7	0.8	0.8
R. Kidney	0.98	0.98	0.98	0.98	0.98	0.7	0.7	0.7	0.7	0.8
Large Bowel	0.89	0.88	0.90	0.87	0.90	1.6	1.6	1.5	2.2	1.3
Liver	0.98	0.98	0.98	0.98	0.98	1.0	1.0	0.8	0.9	0.8
Spinal Canal	0.96	0.96	0.96	0.96	0.95	0.5	0.5	0.5	0.6	0.6
Spleen	0.97	0.97	0.98	0.98	0.98	0.9	0.9	0.9	0.9	0.9
Stomach	0.91	0.91	0.92	0.92	0.92	2.2	2.6	2.4	2.1	2.2
**Mean**	**0.93**	**0.93**	**0.94**	**0.93**	**0.94**	**1.4**	**1.5**	**1.4**	**1.5**	**1.7**

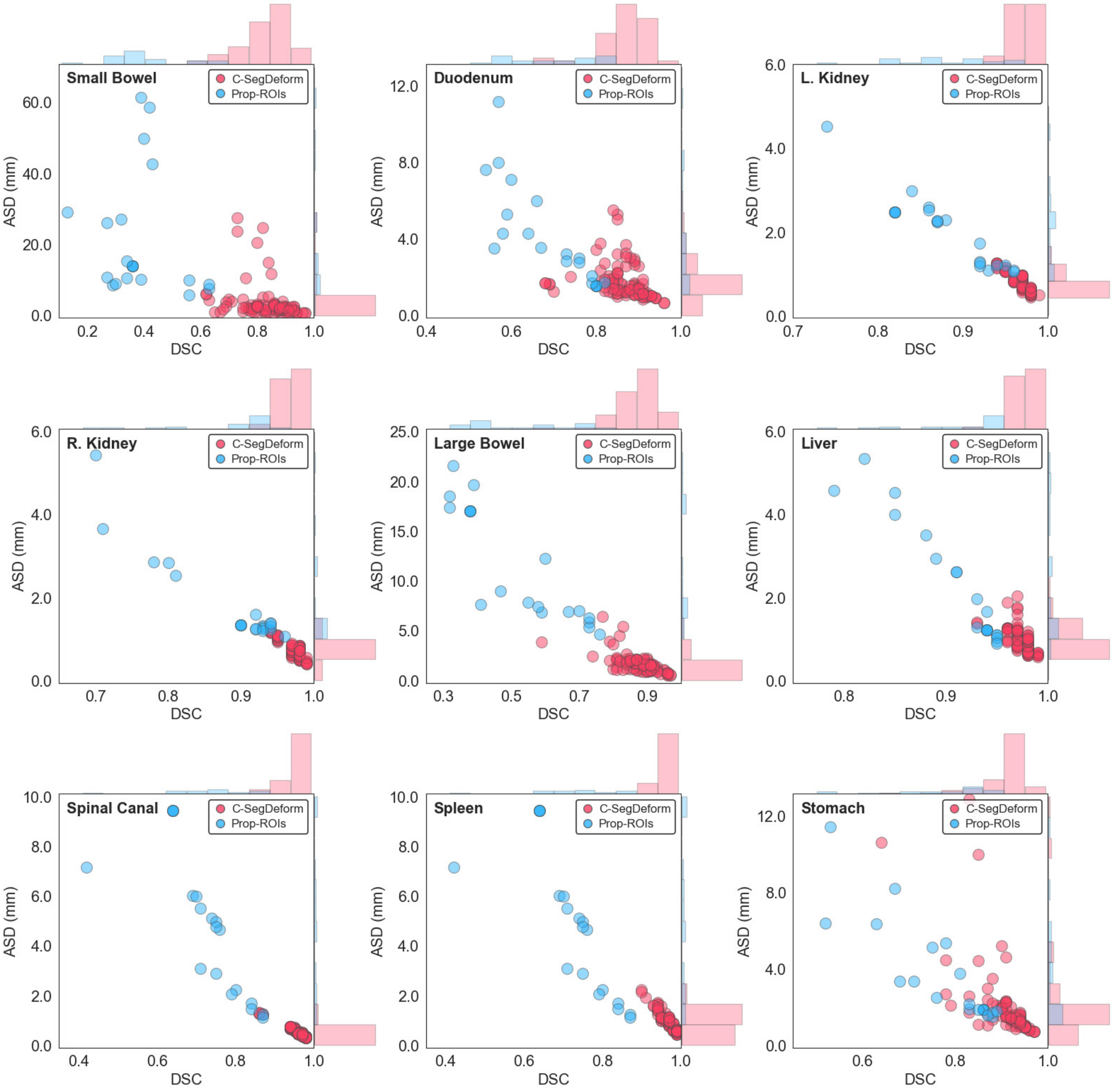

Figure S3: *Comparison between the contours from C-SegDeform and Prop-ROIs with DSC on the x-axis and ASD on the y-axis for each OoI. C-SegDeform's outputs are tightly clustered in the optimal performance region (high DSC values and low ASD). This concentrated distribution indicates that the contours generated by C-SegDeform closely match the ground-truth.*

C.2 Ensemble Learning

We investigated an ensemble approach where contours for a target fraction (e.g., I_5) were generated from all available prior fractions (I_1, I_2, I_3, I_4) using ensemble learning. This ensemble approach was based on the hypothesis that integrating multiple predictions could mitigate outlier errors and improve robustness to atypical anatomical variations present in single fractions. For this purpose, we selected the single best-performing fold model (based on validation performance) and used it for all the predictions.

Table S4 reveals that the ensemble results were very similar to those obtained using only the first fraction as input, suggesting that incorporating information from multiple fractions did not significantly improve accuracy. This aligns with our original aim, i.e., to generate accurate contours for future fractions from a single initial image and contour set, without requiring patient-specific training or additional processing, such as selecting different fraction images for conditioning. Therefore, our proposed approach, with fewer modifications and processing steps than current methods, offers a more efficient alternative that could help reduce clinical workload.

Table S4: *Comparison of the geometric accuracy results between the ensemble learning approach and using only the first fraction I_1. The ensemble learning technique aggregates predictions from multiple prior fractions (e.g., I_1, I_2, I_3) to generate contours for target fractions (I_4 and I_5). However, the results indicate that this approach does not significantly improve performance compared to using only the first fraction as input.*

Organs	DSC		ASD [mm]	
	First Fraction	Ensemble	First Fraction	Ensemble
Small Bowel	0.83	0.85	6.1	5.8
Duodenum	0.89	0.89	1.5	1.8
L. Kidney	0.97	0.97	0.7	0.7
R. Kidney	0.97	0.97	0.7	0.7
Large Bowel	0.90	0.92	1.3	1.2
Liver	0.98	0.98	0.9	0.9
Spinal Canal	0.96	0.96	0.5	0.5
Spleen	0.97	0.97	0.9	0.9
Stomach	0.93	0.92	1.5	2.1
**Mean**	**0.94**	**0.94**	**1.6**	**1.6**

References

[1] F. Isensee et al. ‘nnU-Net: a self-configuring method for deep learning-based biomedical image segmentation’. In: *Nat Methods* 18.2 (2021), pp. 203–211. DOI: 10.1038/s41592-020-01008-z.
